# Elucidating causal effects of type 2 diabetes on ischemic heart disease from observational data on middle-aged Swedish women: a triangular analytical approach

**DOI:** 10.1038/s41598-021-92071-9

**Published:** 2021-06-15

**Authors:** Kristina Sundquist, Sven-Erik Johansson, Ashfaque A. Memon, Susanna Calling, Henrik Ohlsson, Robert Szulkin, Eladio Jimenez, Jan Sundquist

**Affiliations:** 1 Center for Primary Health Care Research, Department of Clinical Sciences Malmö, Lund University, Lund, Region Skåne Sweden; 2 Scandinavian Development Services, Danderyd, Sweden; 3 Department of Preventive Medicine and Public Health, School of Medicine, University of Granada, Granada, Spain; 4 CIBERESP Spain, Chair of Teaching and Research in Family Medicine, SEMERGEN-University of Granada, Granada, Spain

**Keywords:** Cardiovascular diseases, Diabetes, Epidemiology

## Abstract

The association between type 2 diabetes (T2D) and ischemic heart disease (IHD) is well established but the potential causal association needs further studying. In an attempt to elucidate the causal effect of T2D on IHD, we used three different analytical approaches in two different datasets. A well-defined cohort of 6047 women aged 50–59 years were included at baseline (1995 to 2000) and followed until 2015 for IHD. The median follow-up was 16.3 years. We used a Marginal Structural Cox model (MSM Cox) to account for time-varying exposure (time at onset of T2D) and for ten confounders (using inverse probability weighting, IPW). We also compared the MSM-Cox models with traditional Cox regression modelling in the cohort. Finally, we analyzed information on individuals from Swedish population-based registers with national coverage in a comprehensive co-relative design and extrapolated the results to MZ twins. The Hazard Ratio (HR) for IHD in relation to T2D at baseline and T2D occurring during the follow-up in the MSM Cox model weighted by IPW (based on the ten included confounders) was 1.43 (95% confidence interval [CI] 1.07–1.92). The corresponding HR from the traditional Cox regression model was of similar effect size. The average extrapolated MZ twin estimate from our co-relative model was 1.61 (95% CI 1.48–1.86). Our findings, based on a triangular approach, support the existence of a causal association between T2D and IHD and that preventive long-term measures in order to avoid or postpone IHD should include monitoring and treatment of both the T2D itself as well as other cardiovascular risk factors.

## Introduction

The association between type 2 diabetes mellitus (T2D) and ischemic heart disease (IHD) has been demonstrated in both men and women in several observational studies^1–3^. However, the potential causality behind this association has not yet been firmly established. This is mainly because most patients with T2D also suffer from other conventional risk factors for cardiovascular diseases, which may act as potential confounders in the analysis. It is therefore important to adjust for these risk factors although it is often difficult to achieve “full” adjustment; residual confounding has therefore often remained an unsolved problem in most studies. In addition, there is no strong effect of the level of fasting blood glucose on future IHD in patients with T2D and this is also the case among healthy persons^4^. Studies elucidating the potential causality behind the association between T2D and IHD are therefore highly needed for both men and women in order to develop precise preventive measures. In this study, the main focus will be on women as we will take advantage of a highly comprehensive longitudinal sample of middle-aged women called the Women’s Health in the Lund Area (WHILA) with a long follow-up period; this sample has been used in several previous studies on various health outcomes^5^.

Previous literature focusing on women with T2D have suggested that the gender difference in cardiovascular morbidity observed between non-diabetic men and women diminishes when comparing men and women with T2D^2^. The Copenhagen heart study showed that women aged 55–64 years with T2D have a three to fourfold increased risk of having an incident myocardial infarction independent of other known risk factors for cardiovascular diseases^1^. A Finnish study found that women with T2D without prior IHD compared with non-diabetic women with prior IHD had a hazard ratio (HR) of 3.5 (1.8–6.8) for IHD mortality^6^. A meta-analysis, based on 102 studies, showed a HR of 2.59 (2.29–2.93) for IHD among women with diabetes, after adjusting for conventional risk factors (smoking, body mass index, systolic blood pressure, and triglycerides)^4^.

The present study will add to the previous literature because we will have access to a comprehensive set of several potential confounders in the analysis, which will minimize the problem with residual confounding. In an attempt to elucidate the potential causal effect of T2D on IHD, we will use three different approaches in two different datasets: (1) Marginal Structural Modeling (MSM), including inverse probability weighting (IPW) where the exposure is allowed to vary with time (using the WHILA sample); (2) a family-based study design (using information from the entire Swedish female population) to adjust for familial confounding; and (3) A traditional Cox regression model (using the WHILA sample). The use of three different approaches to address one research question, i.e., triangulation, where each approach has its own assumptions, strengths and weaknesses^7^ will make our findings less likely to be artefacts.

## Methods

The first approach in this study is based on the WHILA population, which includes a population-based sample of middle-aged women aged 50–59 years at baseline; the sample has been widely used in several previous studies^8,9^. From Dec 1995 to Feb 2000, a total of 6917 women (out of 10,766 eligible women, i.e., the total population of women in 1995 in five municipalities in southern Sweden, where 3849 women were non-responders) underwent a physical examination and answered a questionnaire^5,10^. In the present study, 6047 women without previous IHD were included. Exclusion of participants was also performed because of missing values in at least one variable. Written informed consent was given by all the participants in the study after full explanation of the purpose and nature of all procedures. The Regional Ethical Review Board in Lund approved the present study (Ref nos. 2011/494, 2015/6 and 2012/795). All methods were performed in accordance with relevant guidelines and regulations.

### Exposure variable

T2D was retrieved from the nationwide Hospital Discharge Register that is maintained by the National Board of Health and Welfare. Type 2 Diabetes Mellitus (T2D) was defined based on the International Classification of Diseases (ICD10: E11; ICD8/9: 250). We used two different exposure variables, one dichotomous variable reflecting T2D onset (Yes/No, 1/0) and one variable categorized with regard to duration of disease into four groups: 0 years (no diabetes); 0.1–5 years, 5.1–10 years and ≥ 10 years. Both these variables were allowed to vary with time. For example, the dichotomous exposure variable for a woman who was diagnosed with T2D 3 years after baseline would be coded as unexposed (0) during the first 3 years and then exposed (1) in the remainder of the follow-up period.

### Outcome variable

We assessed the incidence of the outcome ischemic heart disease (IHD) for the entire sample. The women were followed from the day of screening (baseline) until first hospitalization of IHD or until the end of the study on May 31st 2015. The median follow-up time was 16.3 years. IHD was obtained based on the codes I20–I25 (ICD-10) or 410–414 (ICD-8/9) from the Hospital Discharge Register.

### Potential confounders

In total ten confounders, of which three were ratio variables, were assessed at baseline:

*Age_tv* age was used as a continuous and time-varying variable.

*Education* was dichotomized into low/middle vs. high (university).

*Body mass index (BMI;* (weight (kg)/height^2^ (m^2^)) was categorized into underweight (< 18.5), normal weight (18.5–24.9), overweight (25.0–29.9) and obesity ($\ge$ 30).

*Waist hip ratio (WHR)* was dichotomized into < 0.78 and ≥ 0.78. This cut-off point was chosen based on the characteristics of the study sample^8^.

*Non-fasting blood glucose (B_glu)* was included as a continuous variable. Non-fasting blood glucose has previously been shown to be an independent risk factor for IHD^11,12^.

*Triglycerides (Trig)* was included as a continuous variable.

*Ratio total cholesterol to high density lipoprotein cholesterol (Tc_HDL-C)* was treated as a continuous variable. This ratio has previously been shown to be a strong predictor of IHD^13^.

*Blood pressure* was categorized into three levels, based on the distribution: (1) systolic blood pressure < 140 mmHg and diastolic blood pressure < 90 mmHg, (2) systolic blood pressure 140–149 mmHg or diastolic blood pressure 90–99 mmHg, and (3) systolic blood pressure ≥ 150 mmHg or diastolic blood pressure ≥ 100 mmHg. In the calculation of IPW the *systolic and diastolic blood pressures* were included as continuous variables.

*Fitness* was self-reported and based on the survey question “How physically active are you and how much strenuous exercise do you engage in in your leisure time and in your home?”. The participants’ responses were based on a 7-level ordinal scale from “Basically nothing” to “Regular strenuous activity several times a week”. The variable was categorized into three groups: Poor (level 1–3), Middle (level 4–5), and Good (level 6–7).

*Smoking* was categorized into (1) non-smoker (2) former smoker, and (3) daily smoker.

### Statistical methods

We assessed the incidence of T2D (yes/no), taking the follow-up time (from baseline to occurrence of incidence of T2D) into account. T2D cases diagnosed before baseline were also considered. To minimize the problem with residual confounding and take into account the duration of T2D, we used Marginal Structural Modeling (MSM), including inverse probability weighting (IPW), where the exposure is allowed to vary with time. Robins showed that the parameters of MSM can be estimated using IPW to correct both for confounding and for certain forms of selection bias such as informative censoring^14^. Conceptually, IPW attempts to fully adjust for measured confounders by balancing the confounders across levels of treatment with a treatment weight. It creates a pseudo-population in which all measured confounders are balanced between the different treatment groups. In our case, IPW was used to more robustly assess the causal effects of T2D on IHD. We used the statistical software R^15^ to fit an MSM-Cox to our data, which was split into discrete time-bands (i.e. a counting process structure of the data). The application of this model requires two steps: (1) calculating IPW:s and (2) applying the IPW:s as weights in a Cox regression model with time-varying exposure. We used the *ipw* R-package^16^ to estimate IPW weights for longitudinal data, i.e. the inverse of the probabilities of being exposed (also called a propensity score) to T2D at each time point during the follow-up. To estimate the IPW:s, the following confounders were used: age, education, BMI, WHR, non-fasting blood glucose, ratio total cholesterol/HDL-C, triglycerides, blood pressure (systolic and diastolic), fitness, and smoking. Furthermore, for the dichotomous exposure, a survival model was used to estimate the IPW:s (time from start of study to exposure from T2D) and for the categorized exposure we used a multinomial regression model. The calculated IPW:s were then used as weights, to adjust for confounding, in a Cox regression model with the time-varying exposure and a robust standard error (SE).

The MSM Cox model estimates a marginal hazard ratio (HR), which has a causal interpretation if three key assumptions are fulfilled: (1) most important confounders are included; (2) “Positivity: The positivity assumption states that every individual must have at least a non-zero probability to be exposed to T2D. This means that none of the predicted values that are used to compute the propensity score (and the IPW) can be 0 or 1.”^15^; (3) Correct specification of the IPW model^17^. We included the most important confounders (in total ten) and thus judge that we have correctly specified the IPW model. Regarding assumption 2, all women had a theoretical probability to be exposed to T2D. Since the size of some of our weights was very large, this could be a sign of a violation of the positivity assumption (assumption 2). This was handled by truncation of the weights as described by Cole et al.^18^. IPW is estimated using a Cox model that regresses covariates to a treatment group (exposure) variable. IPW is time-varying. We used a marginal approach and weights to balance the confounders across treatment exposure levels. In this study, T2D is treated as a time-varying exposure. It is also a binomial variable (onset of T2D during follow-up or present at baseline). MSMs are used to estimate the causal *average treatment effect* (*ATE*)^19^ from observational data. As an example, the HR for the dichotomous exposure (T2D: yes/no) should be interpreted as the ratio between the IHD hazard when the study population is exposed (T2D) and the IHD hazard when the same population is unexposed during the whole follow-up period^17^.

However, as previously stated, this interpretation is valid under certain assumptions, i.e. minimal unmeasured confounding. Thus, IPW can provide unbiased estimates of marginal causal effects in the context of confounding. In the ideal case, we should take into account all possible confounders. In this study we have included the most important confounders based on previous studies and by our judgement.

We also compared the MSM-Cox models with ordinary Cox regression models, adjusted for the same ten confounders as included in the IPW. We used the statistical software R^15^ for analyses and STATA^20^ for data processing.

### Family design using the Swedish population registers

In the second approach we analyzed information on individuals from Swedish population-based registers with national coverage. These registers were linked using each person’s unique identification number replaced by a serial number to preserve confidentiality. Our database for the family design analysis was created by selecting all women born in Sweden from 1945 to 1965 (n = 2,375,535). These individuals were linked with the Swedish medical registers where we selected the first registration of IHD (ICD10: I20-I25 and ICD8/9:410-414), and the first registration of T2D (ICD10: E11) for each individual. In the database we also included date of emigration, date of death or end of follow-up period (2016-12-31). From this database we created 16 different cohorts where the follow-up for IHD for the 16 cohorts started January 1st every year from 2001 to 2016. This means that for the first cohort a woman must be recorded with T2D before January 1st, 2001, in order to be included in the T2D-group and is not allowed to have had a record of IHD before January 1st, 2001. The next cohort was defined in the same way but 1 year later (recorded with T2D before January 1st, 2002) in order to be included in the T2D-group, and not recorded with an IHD registration before January 1st, 2002. The cohorts are similar, but specific individuals change group from non-T2D to T2D, and/or disappears from the cohort if she dies or gets a diagnosis of IHD during the year. Also, the number of years of follow-up changes across cohorts. For each cohort we performed a Cox regression model with T2D (yes/no) as exposure and time to IHD, emigration, death or end of follow-up (which ever came first) as outcome. The models were adjusted for year of birth. In the next step we used a co-relative design. In a co-relative design, we can examine if the results from the entire population reflect confounding by unmeasured familial (environmental and genetic) risk factors. From the Swedish Multi-Generation, we identified all full- and half-sibling and cousin pairs (born within between 1945 and 1965). Using stratified Cox proportional hazards models, with a separate stratum for each relative pair, we refitted the analyses for the 16 different cohorts. The Hazard ratio (HR) was then adjusted for a range of unmeasured genetic and environmental factors shared within the relative pair. Full-siblings share 50% of their genes and a large part of environmental factors suggesting that the HR for full-siblings is partly controlled for confounding by genes and shared environment. Half-siblings and cousins share, respectively, on average 25% and 12.5% of their genes identical by descent. Finally, for each cohort, we combined all four samples (i.e., population, full-siblings, half-siblings and cousins) into one dataset in which we modelled the association between T2D and IHD with two parameters: one main effect and one as a linear function of the genetic resemblance; i.e., 0 for the population, 0.125, for the cousin, 0.25 for the half-sibling, and 0.5 for the full-siblings. The HR for the second parameter gives an indication of the size of the familial confounding. We also obtained an improved estimation of the association among all relatives, but mainly we could extrapolate to a situation where we have complete genetic similarity (i.e., pseudo MZ-twin estimates).

From our analyses we obtained 16 HRs for the effect of T2D on IHD in the population samples, and 16 HRs for the effect of T2D and IHD controlled for all shared genetic and familial environmental factors (pseudo MZ-twin estimates). Thereafter, we used a meta-analysis to calculate one average HR for the population and one average HR for the pseudo MZ-twin estimates.

### Ethics approval and consent to participate

The Regional Ethical Review Board in Lund approved the study (Ref nos. 2011/494, 2015/6 and 2012/795). All methods were performed in accordance with relevant guidelines and regulations. Written informed consent was given by all the participants in the WHILA study.

## Results

**Figure 1:**
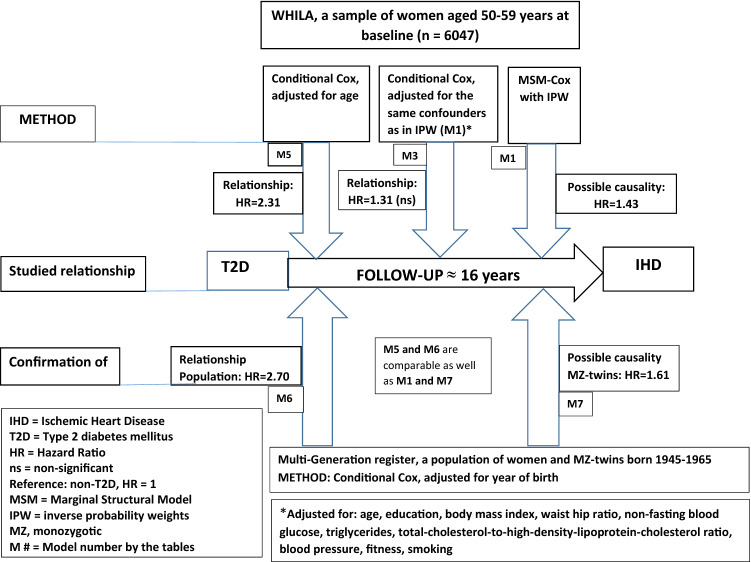
The two study populations and an overview of the methods.

Table 1 shows a description of the chosen confounders in the WHILA cohort by the following four sub-groups: free of both IHD and T2D, prevalent T2D at baseline, incident T2D during the follow-up, and incident IHD during the follow-up. Women with diabetes had lower education, larger WHR and BMI, higher blood pressure, and poorer fitness than those free of T2D and IHD. The prevalence of obesity was 10.6% in women without T2D, 36.3% in women with prevalent T2D and 34.1% in women with incident T2D. See also Fig. 1 for an overview.

**Table 1:** Description (means and percentages) of confounders in four sub-groups, n = 6047.

Variable	Free of IHD and T2D	Prevalent-T2D	Incident-T2D	IHD
**n**	4925	170	552	537
**Means**				
Duration of T2D (years)	–	8.3 (med = 4)	8.5 (med = 7.6)	–
Age (years)	56.3	57.3	56.9	57.0
Ratio ttotal chol./HDL-C	3.6	4.1	4.3	4.1
Blood glucose (mmol/l)	6.0	9.7	6.8	6.4
Triglycerides (mmol/l)	1.6	2.2	2.1	2.0
BMI (units)	25.0	28.2	28.2	25.9
Systolic blood pressure (mmHg)	131	136	140	136
Diastolic blood pressure (mmHg)	85	85	89	86
**Distribution (%)**				
Education				
Low/middle	62.4	79.1	74.6	71.8
High	37.6	20.9	25.4	28.2
Waist hip ratio (cm)				
≤ 0.78	59.5	25.2	28.0	56.3
> 0.78	40.5	74.8	72.0	43.7
BMI (kg/m^2^)				
Underweight (< 18.5)	1.2	0.7	0.2	1.7
Normal (18.5–24.9)	54.2	29.7	26.6	42.5
Overweight (25.0–29.9)	34.0	33.4	39.1	38.9
Obesity (≥ 30.0)	10.6	36.3	34.1	16.9
Blood pressure (mmHg)				
SBP < 140 and DBP < 90	55.5	41.0	32.3	43.6
SBP = 140–149 or DBP = 90–99	26.1	31.5	31.8	29.8
SBP ≥ 150 or DBP ≥ 100	18.4	27.5	35.9	26.6
Smoking				
No	60.2	61.1	56.7	50.7
Former	19.7	20.6	23.0	22.3
Daily smoking	20.1	18.3	20.3	28.0
Fitness (7-level ordinal scale)				
Poor (1–3)	5.0	8.5	9.8	8.3
Middle (4–5)	51.2	63.1	53.5	57.8
Good (6–7)	43.8	28.4	36.7	33.9

The WHILA cohort. IHD is a subgroup in the total sample.

T2D, type 2 diabetes mellitus; IHD, ischemic heart disease; HDL-C, high-density-lipoprotein-cholesterol; BMI, body mass index; SBP, systolic blood pressure; DBP, diastolic blood pressure.

Table 2 shows the HRs for IHD in relation to T2D status during follow-up in an MSM Cox model (M1) (based on the ten included confounders) and an MSM multinomial model (M2). We found a HR of 1.43 (95% CI 1.07–1.92) for IHD comparing T2D exposure with no exposure in model M1. In the MSM multinomial model (M2) based on the duration of T2D categorized into three levels, there was an apparent gradient showing an increasing risk of IHD by increasing duration of T2D where no T2D was used as reference level (HR = 1): 0.1–5 years HR = 0.83 (95% CI 0.44–1.55); 5.1–10 years HR = 1.50 (95% CI 0.87–2.59); > 10 years HR = 1.99 (95% CI 1.33–2.97).

**Table 2:** Hazard ratios (HRs, 95% CI) for the association between type 2 diabetes (T2D) and ischemic heart disease (IHD), estimated from two MSM models with IPW, ten included confounders.

Model 1 (M1)		Model 2 (M2)	
MSM Cox		MSM multinomial model	
Test of possible causality^a^		Duration of T2D^a^	
Level	HR (95% CI)	Level	HR (95% CI)
No diabetes	1 (reference)	No diabetes	1 (reference)
T2D (yes/no)	1.43 (1.07–1.92)	0.1– < 5 years	0.83 (0.44–1.55)
		5.1–10 years	1.50 (0.87–2.59)
		> 10 years	1.99 (1.33–2.97)

HRs shown with 95% CI of ischemic heart disease in different models.

Included confounders: age, education, body mass index, waist hip ratio, non-fasting blood glucose, triglycerides, total-cholesterol-to-high-density-lipoprotein-cholesterol ratio, blood pressure, fitness, smoking.

MSM, Marginal Structural Model; IPW, inverse probability weighting; HR, hazard ratio; CI, confidence interval; T2D, type 2 diabetes mellitus.

^a^Weights truncated at 2 and 98%.

Table 3 shows the corresponding HRs from the traditional Cox regression, adjusted for the same confounders as included in the models in Table 2. The HR was for M3 (comparable with M1 in Table 2) 1.31 (95% CI 0.99–1.73). The HRs for M4 (comparable with M2 in Table 2) were as follows: 0.1–5 years: HR = 0.75 (95% CI 0.43–1.32), 5.1–10 years: HR = 1.19 (95% CI 0.75–1.91), and > 10 years: HR = 2.02 (95% CI 1.39–2.93).

**Table 3:** Hazard ratios (HRs, 95% CI) for the association between type 2 diabetes (T2D) and ischemic heart disease (IHD), estimated from conditional Cox regression model in two different models.

Model 3 (M3)		Model 4 (M4)	
Conditional Cox		Conditional Cox	
	HR (95% CI)	Years since onset of T2D	HR (95% CI)
No T2D	1 (reference)	No T2D	1 (reference)
T2D	1.31 (0.99–1.73)	0.1– < 5 years	0.75 (0.43–1.32)
		5.1–10 years	1.19 (0.75–1.91)
		> 10 years	2.02 (1.39–2.93)

Traditional Cox models adjusted for the same confounders as included in the Marginal Structural Models (age, education, body mass index, waist hip ratio, non-fasting blood glucose, triglycerides, total-cholesterol-to-high-density-lipoprotein-cholesterol ratio, blood pressure, fitness, smoking) and shown as HRs with 95% CI of ischemic heart disease.

HR, hazard ratio; CI, confidence interval; T2D, type 2 diabetes mellitus.

Table 4 shows a comparison of the results from the two different study populations and includes the population estimates and the extrapolated MZ twin estimates based on the family design using the Multigeneration register from the entire Swedish population. In the population estimate the average HR was 2.70 (95% CI 2.58–2.82), suggesting that women with T2D had 2.7 times higher risk for IHD than women without T2D. This estimate is similar to the one obtained in the Cox regression model adjusted for age in the WHILA sample and well within the 95% confidence intervals (HR_whila_ = 2.31; 95% CI 1.61–3.31). The average extrapolated MZ twin estimates from our co-relative model was 1.61 (95% CI 1.48–1.86). That is, this model predicts, in a pair of MZ twins, a 61% greater risk for IHD in the twin with T2D versus in the twin with no T2D. The results indicate that the effect of T2D could partially be attributed to familial factors (genetic and environmental) shared by MZ-twins.

**Table 4:** Comparison of the results (M6 vs. M5 and M7 vs. M1, respectively) for the hazard ratios (HRs, 95% CI), from the two study populations.

The multigeneration registry	Model	HR (95% CI) of IHD among those with T2D compared with non-T2D (HR = 1, ref)
Population estimate^a^	M6	2.70 (2.58; 2.82)
MZ twins estimate^a^	M7	1.61 (1.48; 1.86)

WHILA		HR (95% CI) of IHD among those with T2D compared with non-T2D (HR = 1, ref)
Age-adjusted conditional Cox	M5	2.31 (1.61–3.31)
MSM-Cox^b^	M1	1.43 (1.07–1.92)

HR, hazard ratio; CI, confidence interval; IHD, ischemic heart disease; T2D, type 2 diabetes mellitus; MZ, monozygotic; MSM, Marginal Structural Modeling.

^a^Adjusted for year of birth.

^b^Adjusted for age, education, body mass index, waist hip ratio, non-fasting blood glucose, triglycerides, total-cholesterol-to-high-density-lipoprotein-cholesterol ratio, blood pressure, fitness, smoking.

## Discussion

The present study confirmed, using three different approaches, an association between T2D and IHD, which is in line with previous studies^4,21–24^. The novelty of the present study is the use of three different analytical approaches on two different data sources, of which one data source included ten potential confounders. Taken together, this helped us to elucidate causal processes and minimize problems with residual confounding. Causal inferences from observational data should always be considered tentative. However, confidence in the findings could be higher if similar results are obtained from different methods; in our case, we used three methodological approaches, i.e., “triangulation”^7^ of which two (MSM Cox and MZ twin estimates) could be considered to elucidate etiological processes in a more robust manner. Given the similar results from in total three different methodological approaches, we believe that our findings could be considered to be valid. Moreover, many previous studies that assessed the association between T2D and IHD were based on men although previous literature suggests that the predictive risk patterns in women have not been fully studied^25,26^. To the best of our knowledge, no previous study on the potentially causal relationship between T2D and IHD in women has been based on “triangulation”.

A causal association between T2D and IHD has also been suggested by the authors of a Mendelian randomization study of the influence of T2D and fasting glucose on IHD risk. That study was based on data from multiple genetic variants associated with T2D and fasting glucose^27^. Another Mendelian randomization study showed that the risk of IHD increased stepwise with increasing non-fasting glucose levels^28^.

Our findings of an apparent gradient between increasing duration of T2D and a higher risk of IHD agreed with a Swedish national register study from 2019 that found that T2D patients without IHD had an almost linear association between age at start of glucose lowering drugs and IHD risk^29^. A dose–response relationship further supports a causal association as well as our findings obtained from the Marginal Structural Modeling (MSM), where the exposure is allowed to vary with time. To the best of our knowledge, this is the first study to use MSM to analyze the potentially causal relationship between T2D and IHD in middle-aged women. Our MSM using IPW included ten important confounders in the sample of 6047 women and suggested a 43% higher risk for IHD among those women with T2D. Our results from an ordinary Cox regression model with the same ten confounders produced similar results with a 31% higher risk of IHD among the women with T2D. Finally, the use of a co-relative design with 2,375,535 females from the Swedish population registers is also a strength.

There are also some limitations with our study. Firstly, the non-response rate of 35% may result in selection bias where those who participated in the study may be healthier than those who did not^30^. However, non-response analysis has shown that non-responders and responders have an equal risk of IHD^9^. Secondly, although we included ten important confounders, we cannot fully rule out the possibility of residual confounding. For example, we had no information on the severity of the diseases, how well controlled they were or lipid lowering medication; however, during the study period statins were not recommended as primary prevention of IHD in Sweden. Thirdly, some variables (education, fitness and smoking) were self-reported although most of the included variables were based on clinical assessments from the physical examinations. However, the large sample size, the triangular methodological approach and the consistency of our results with those of other studies support the validity of our findings. In addition, the study population in WHILA seems to be highly representative. For example, the obesity rates are in line with those reported in previous studies^31^.

The clinical implications of the present study are many. For example, we found that the women with T2D had worse results in almost all clinical and lifestyle measurements. The ratio of total cholesterol/HDL-C was much higher in both those with previous T2D and incident T2D than their non-diabetic counterparts, and the triglyceride levels, BMI and WHR were also higher among the women with T2D whereas the levels of exercise were lower. The onset of T2D may in many cases occur years before the diagnosis, and the poor cardiovascular risk profile in the present study population emphasizes the importance of identifying individuals with T2D at an earlier stage in order to start medication and lifestyle advice. The high proportion of obesity in women with prevalent T2D at baseline (36.3%) reflects the metabolic disorder in women of this age group and this proportion may even be higher in some populations^32^. However, there were no differences in smoking between the women with T2D and the controls, which is in agreement with previous studies showing that the prevalence of smoking among people with T2D appears to be similar to that of the general population^33^. However, it should still be particularly important to include smoking cessation, in addition to other preventive measures and lifestyle interventions, in women with T2D considering their elevated risks of cardiovascular diseases.

## Conclusions

Our findings support the existence of a causal association between T2D and IHD and that preventive long-term measures in order to avoid or postpone IHD should be broad and include monitoring and treatment of the T2D itself as well as other risk factors.

## Abbreviations

BMI: Body mass index
CI: Confidence interval
HDL-C: High density lipoprotein cholesterol
HR: Hazard ratio
ICD: International Classification of Diseases
IHD: Ischemic heart disease
IPW: Inverse probability weighting
MSM: Marginal Structural Modeling
MZ: Monozygotic
T2D: Type 2 diabetes mellitus
WHILA: Women’s Health in the Lund Area
WHR: Waist hip ratio
